# Dovitinib preferentially targets endothelial cells rather than cancer cells for the inhibition of hepatocellular carcinoma growth and metastasis

**DOI:** 10.1186/1479-5876-10-245

**Published:** 2012-12-10

**Authors:** Zhi-Yuan Chen, Ming Shi, Li-Xia Peng, Wei Wei, Xin-Jian Li, Zhi-Xing Guo, Shu-Hong Li, Chong Zhong, Chao-Nan Qian, Rong-Ping Guo

**Affiliations:** 1 Department of Hepatobiliary Surgery, Sun Yat-sen University Cancer Center, 651 Dongfeng Road East, Guangzhou, 510060, China; 2 State Key Laboratory of Oncology in South China, Sun Yat-sen University Cancer Center, 651 Dongfeng East Road, Guangzhou, 510060, People’s Republic of China; 3 Laboratory of Cancer and Developmental Cell Biology, Van Andel Research Institute, 333 Bostwick Ave. NE, Grand Rapids, MI, 49503, USA; 4 Department of Neuro-Oncology, University of Texas MD Anderson Cancer Center, 6767 Bertner Ave., Unit 1002, BSRB S5.8116, Houston, TX, 77030, USA; 5 Department of Hepatobilliary Surgery, 1st Affiliated Hospital to Guangzhou University of Chinese Medicine, 16 Jichang Road, Guangzhou, 510405, China

**Keywords:** Dovitinib, Endothelial cells, Hepatocellular carcinoma, Tumor growth, Tumor metastasis

## Abstract

**Background:**

Dovitinib is a receptor tyrosine kinase (RTK) inhibitor targeting vascular endothelial growth factor receptors, fibroblast growth factor receptors and platelet-derived growth factor receptor β. Dovitinib is currently in clinical trials for the treatment of hepatocellular carcinoma (HCC).

**Method:**

In this study, we used five HCC cell lines and five endothelial cell lines to validate molecular and cellular targets of dovitinib.

**Results:**

Tumor growth and pulmonary metastasis were significantly suppressed in an orthotopic HCC model. Immunoblotting revealed that among known dovitinib targets, only PDGFR-β was expressed in two HCC cell lines, while four of five endothelial lines expressed PDGFR-β, FGFR-1, and VEGFR-2. Dovitinib inhibited endothelial cell proliferation and motility at 0.04 μmol/L, a pharmacologically relevant concentration; it was unable to inhibit the proliferation or motility of HCC cells at the same concentration. Immunohistochemical analyses showed that dovitinib significantly decreased the microvessel density of xenograft tumors, inhibiting proliferation and inducing apoptosis in HCC cells.

**Conclusion:**

Our findings indicate that dovitinib inhibits HCC growth and metastasis preferentially through an antiangiogenic mechanism, not through direct targeting of HCC cells.

## Introduction

Hepatocellular carcinoma (HCC) is characterized by highly vascularized and rapid tumor progression, a high recurrence rate after surgical resection, and an extremely poor prognosis. It is the fifth most common cancer in the world, and the third most frequent cause of cancer death [1-4]. The highly vascularized nature of HCC has been considered as the main reason for its devastating outcome, because of intrahepatic and distant metastases [5-7]. Vascular endothelial growth factor (VEGF), basic fibroblast growth factor (bFGF), and platelet-derived growth factor (PDGF) are three important pro-angiogenic factors involved in hepatocarcinogenesis, and they participate in the neovascular, invasive, and metastatic potentials of HCC [8-11].

VEGF expression is detected in dysplastic nodules and correlates with histological grades; VEGF is increased during hepatocarcinogenesis [8]. Sorafenib, an inhibitor of several kinases, including Raf-1 and VEGF receptor (VEGFR), is currently the first-line therapy for advanced or recurrent HCC. It has a modest survival benefit, but patients develop subsequent drug resistance [12-14].

Dovitinib (TKI258; formerly CHIR258) is a potent inhibitor of receptor tyrosine kinases (RTKs). It inhibits VEGFR-1, VEGFR-2, and VEGFR-3; fibroblast growth factor receptors (FGFR1, FGFR2, and FGFR3); and platelet-derived growth factor receptor β (PDGFR-β) [15,16]. Dovitinib is reported to directly inhibit the proliferation and survival of colon cancer and leukemia cells, which harbor either activating mutations or translocations in the target RTKs or their ligands, at pharmacologically relevant concentrations of 0.01–0.3 μmol/L [17-19].

In preclinical studies, dovitinib has been able to inhibit xenograft HCC growth in immunodeficient mice and even overcome sorafenib resistance [20,21]. However, the lack of somatic mutations of RTK genes in HCC has caused doubt about whether HCC cells are the primary cellular target of dovitinib [22]. It has been reported that endothelial cells and perivascular cells (pericytes) can express VEGFR, PDGFR, and/or FGFR [8,23,24]; thus, these cells are theoretical targets of dovitinib, and the drug might act as an angiogenesis inhibitor in vivo. However, the ability of dovitinib to suppress tumor angiogenesis has not been established.

In the present study, our aim was to reveal the cellular targets of dovitinib in HCC treatment at pharmacologically relevant concentrations, which is crucial for the future development of this treatment strategy.

## Materials and methods

### Kinase inhibitor

Dovitinib (CHIR-258, TKI258) [4-amino-5-fluoro-3-[6(4-methyl-1-piperazinyl)-1H- benzimidazol-2-yl]-2(1H)-quinolinone], with a molecular weight of 392.4, was provided by Novartis Pharma AG (Novartis Institutes for Biomedical Research, Basel, Switzerland).

### Cells and cell culture

The human HCC cell lines MHCC-97H, QGY-7703, SMMC7721, Hep3B, and CRI2234, as well as a human bone marrow endothelial (HBME) line, were maintained in DMEM or RPMI 1640 (Invitrogen) supplemented with 10% fetal bovine serum (FBS; Invitrogen), 100 IU/mL penicillin, and 100 μg/mL streptomycin (Invitrogen) in a humidified incubator containing 5% CO_2_ at 37°C. Human umbilical vascular endothelial cells (HUVECs), human dermal microvascular endothelial cells (HMVECs), human umbilical artery endothelial cells (HUAECs), and human lung microvascular endothelial cells (HLMVECs) were maintained in Clonetics Endothelial Basal Medium-2 (EBM-2) supplemented with essential growth factor supplements EGM-2 SingleQuots or EGM-MV SingleQuots (Lonza). All the cell lines were used within 50 passages.

### Cell viability assay

Cell viability was assessed using an 3-(4,5-dimethylthiazol-2-yl)-5-(3-carboxymethoxyphenyl)-2-(4-sulfophenyl)-2H-tetrazolium (MTS) assay kit (Sigma) with dye conversion at 490 nm, following the manufacturer’s instructions. Briefly, cells were seeded 3 × 10^3^/well in a 96-well flat-bottomed plate and starved in no serum for 18 h, and were then treated with increasing concentrations of dovitinib and stimulated with 30 ng/mL recombinant human VEGF or PDGF-BB (Sigma V7259 or SRP3138). At 72 h, 20 μl of MTS was added to each well. After 1.5 h of incubation at 37°C, the results were analyzed by a plate reader at 490 nm. The sample data was normalized to background readings of medium only.

### In vitro migration and invasion assays

For Transwell migration assays, 5.0 × 10^4^ HCC cells or endothelial cells in 500 μl of serum-free DMEM or EBM were added to the cell culture inserts with an 8-μm microporous filter without an extracellular matrix coating (Becton Dickinson Labware). To the bottom chamber was added 800 μL of DMEM or EGM containing 10% FBS. After 24 h of incubation, the cells on the lower surface of the filter were fixed, stained, and counted under a microscope (×100 magnification). The number of migrated cells in five random optical fields from triplicate filters was averaged. For invasion assays, the inserts of the chambers to which the cells were seeded were coated with Matrigel (Becton Dickinson Labware). The number of invaded cells in five random optical fields (×100 magnification) was averaged from triplicate inserts.

For the wound healing assay, the cells were plated in 6-well plates (3 × 10^5^ cells/well) and allowed to attach and reach confluence. A scratch was made using a sterile 100-μl pipette tip and detached cells were removed by washing with PBS. Phase contrast images were taken at specified time points. The scratched wound was evaluated at 18 h (endothelial cells) or at 48 h (HCC cells) after scratching.

### Efficacy of dovitinib in an orthotopic HCC model

Male athymic mice between 4 and 5 weeks of age were purchased from Shanghai Institutes for Biological Sciences (Shanghai, China). All the animal studies were conducted in accordance with the principles and procedures outlined in the guidelines of the Institutional Animal Care and Use Committee at Sun Yat-sen University Cancer Center. Mice were anesthetized by continuous inhalation of isoflurane (Baxter Healthcare, NJ).

For orthotopic xenografts, an upper abdominal midline incision was made. MHCC-97H, SMMC7721 or QGY-7703 HCC cells (1 × 10^6^) in 30 μl of culture medium with 33% Matrigel were injected into the left lobe of the liver using an insulin syringe with a 31-gauge needle (Becton Dickinson, NJ). Two weeks later, the nude mice were randomized into three groups of 20 mice each and were treated either with 0.9% sodium chloride or 25 or 50 mg/kg of TKI258 for 14 days. On day 30 after tumor cell inoculation, the animals were weighed, euthanized, and autopsied. The liver and lungs were weighed and sampled for tissue sectioning. To examine lung metastases, 100 sequential lung sections (4 μm) were cut from the lungs of each mouse and every tenth section was stained with hematoxylin and eosin (H&E).

### Immunohistochemistry (IHC)

Formalin-fixed and paraffin-embedded sections 4 μm thick were dewaxed in xylene and a gradient of alcohols, hydrated, and washed in PBS. After pretreatment in a microwave oven (30 min in citrate buffer, pH 6.0), endogenous peroxidase activity was blocked by 0.3% hydrogen peroxide for 10 min, and the sections were incubated with 10% normal goat serum for 15 min. Primary antibodies—rabbit polyclonal anti-CD34 (Santa Cruz Biotechnology, Santa Cruz, CA), rabbit polyclonal anti-Ki67 (Abcam, Cambridge, UK) and rabbit polyclonal anti-PARP (Abcam, Cambridge, UK)—were applied overnight in a moist chamber at 4°C. A standard avidin-biotin peroxidase technique (DAKO, Carpinteria, CA) was applied. Briefly, biotinylated goat anti-rabbit immunoglobulin, goat anti-rat immunoglobulin, and avidin-biotin peroxidase complex were applied for 30 min each, with 15-min washes in PBS. The reaction was finally developed using the DAKO Liquid DAB^+^ Substrate-Chromogen System. The methods for quantification of microvessel density (MVD), proliferation index, and apoptosis index were reported previously [25,26]. Briefly, the largest section of each xenograft tumor was analyzed by randomly capturing images of microscopic fields at low magnification, and the microvessels or stained cells were counted and averaged. The final results were the mean value of each case from two independent referees.

### Immunoprecipitation and immunoblotting

Cells were lysed in cold RIPA buffer (100 mM Tris HCl, 300 mM NaCl, 2% NP40, 0.5% sodium deoxycholate) supplemented with a proteinase inhibitor cocktail (Roche, Indianapolis, IN) and a phosphatase inhibitor cocktail (Merck, Darmstadt, Germany). Protein concentration was determined using a detergent-compatible protein assay according to the manufacturer’s instructions (Bio-Rad). Protein (1 mg) from each sample was immunoprecipitated overnight at 4°C with an anti-VEGFR-2, anti-PDGFR-β, or anti-FGFR-1 (Cell Signaling Technology) antibody plus protein G/A agarose beads (Pierce). Immune complexes were washed with cold RIPA buffer containing proteinase inhibitors and phosphatase inhibitor. Proteins were eluted by boiling in SDS sample buffer, separated by SDS-PAGE, and transferred to polyvinylidene difluoride membrane (Millipore). Membranes were probed with an anti-phosphotyrosine antibody (Cell Signaling Technology) and then stripped with stripping buffer (Abcam). To detect total VEGFR-2, PDGFR-β, and FGFR-1 levels, membranes were re-probed with the same anti-VEGFR-2, anti-PDGFR-β, and anti-FGFR-1 antibody that was used for the immunoprecipitation. Immunoblotting of phospho- ERK1/2 and ERK1/2 (Cell Signaling Technology) was performed on whole-cell lysates (40 μg) with β-actin (Abcam) as a loading control.

### Statistical analysis

Continuous data were expressed as median and range and were compared between groups using one-way ANOVA and Dunnett *t* test. Categorical variables were compared using the chi-square test, or Fisher’s exact test, where appropriate. All data were analyzed using the SPSS 13.0 computer program, and significant difference was defined as *P* < 0.05.

## Results

### Dovitinib inhibited HCC xenograft tumor growth and metastasis

**Figure 1:**
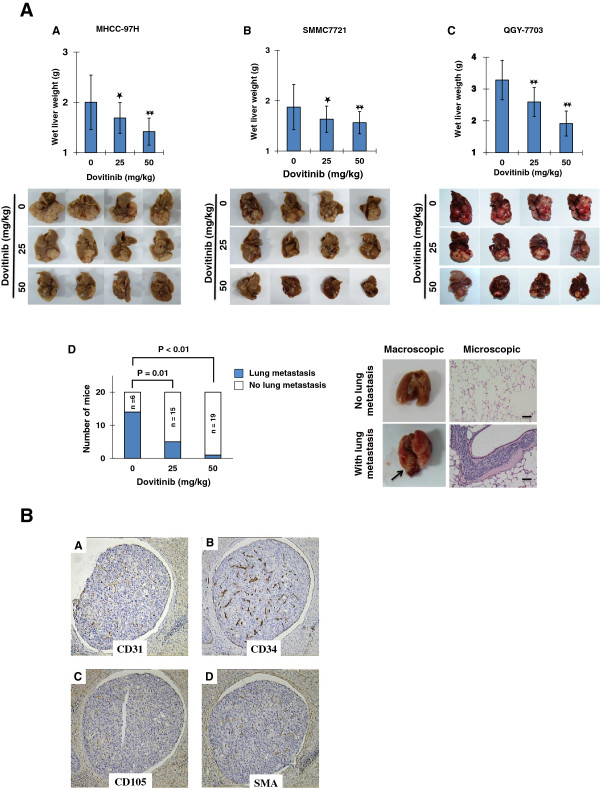
**Dovitinib inhibited the growth and metastasis of HCC in an orthotopic xenograft model.A**) Growth of MHCC-97H xenograft tumors was dose-dependently inhibited by dovitinib. The wet liver weight of 0, 25 and 50 mg/kg were 2.00 ± 0.54, 1.68 ± 0.31 and 1.41 ± 0.27 g, respectively. The four largest liver tumors of each group are shown. **B**) Significant inhibition of SMMC7721 xenograft tumors by dovitinib. The wet liver weight of 0, 25 and 50 mg/kg were 1.87 ± 0.45, 1.63 ± 0.26 and 1.56 ± 0.22 g, respectively. **C**) Dose-dependent inhibition of QGY-7703 xenograft tumors by dovitinib. The wet liver weight of 0, 25 and 50 mg/kg were 3.44 ± 0.72, 2.59 ± 0.45 and 1.91 ± 0.39 g, respectively. **D**) For the mice bearing the high-metastasis HCC cell line MHCC-97H, dose-dependent inhibition of lung metastases by dovitinib was observed under a microscope. The arrow points to macro-metastasis; scale bar, 100 μm. The results in panels A-C are expressed as the mean ± the standard deviation. *black star*, *P* < 0.05; *double black star*, *P* < 0.01.

The therapeutic effect of dovitinib was examined using the orthotopic HCC model. Continuous dovitinib treatment for 2 weeks at doses of 25 or 50 mg/kg was started 14 days after orthotopic injection of MHCC-97H, SMMC7721, or QGY-7703 cells. There was no significant change in body weight of the animals in each treatment group compared with that of the control animals (data not shown). Dovitinib significantly inhibited primary tumor growth in a dose-dependent manner relative to the control group (Figure 1A–C). In MHCC-97H, a HCC cell line with high metastasis capability [27], dovitinib also inhibited pulmonary metastasis in a dose-dependent manner (Figure 1D).

### Dovitinib inhibited RTK signaling pathways in vitro

**Figure 2:**
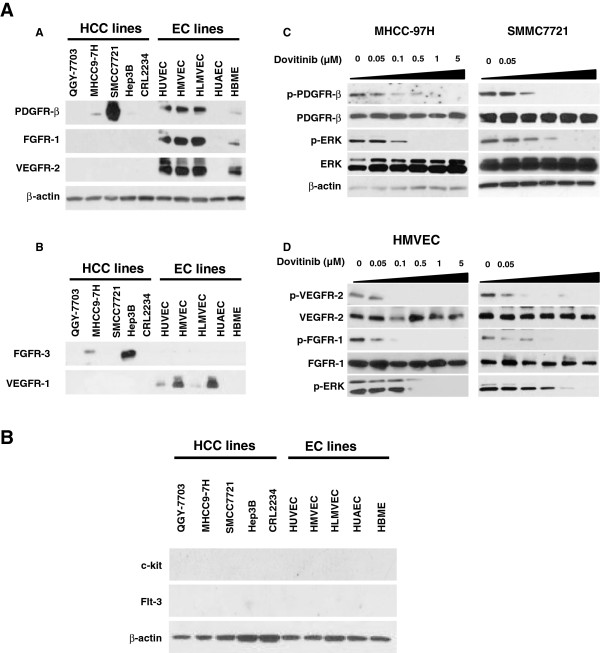
**Expression profile of RTKs and the effect of dovitinib on RTK signaling in HCC and endothelial cells.A**) Expression of PDGFR-β, FGFR-1, and VEGFR-2 in HCC and endothelial cell lines as detected by immunoblotting. **B**) Expression of FGFR-3, and VEGFR-1 in HCC and endothelial cell lines **C**) Phosphorylation of p-PDGFR-β and p-ERK were inhibited by dovitinib at pharmacologically relevant concentrations in MHCC-97H and SMMC7721 cells. **D**) Dovitinib inhibited the phosphorylation of FGFG-1, VEGFR-2, and downstream ERK in HMVEC and HUVEC endothelial cells at pharmacologically relevant concentrations.

Pharmacokinetic and pharmacodynamic studies have revealed that the pharmacologically relevant concentration of dovitinib is 0.01–0.3 μmol/L [17,18]. To evaluate the potential effect of dovitinib at pharmacological concentration on the activation of RTK signaling pathways in vitro, we first examined the expression and activation of VEGFR, FGFR, PDGFR, Flt-3, and c-KIT in HCC cell lines and endothelial cell lines by immunoblotting. FGFR-3 was expressed by Hep3B and MHCC-97H, VEGFR-1 was expressed by two endothelial cells (Figure 2B), PDGFR-β was clearly expressed by two cell lines, MHCC-97H and SMMC7721. In contrast, four of the five endothelial cell lines homogenously expressed VEGFR-2 and FGFR-1 (Figure 2A). Flt-3 and c-KIT were undetectable in all cell lines (Additional file 1: Figure S1).

Based on the combined data of the mice and the cell lines, we focused our study on VEGFR-2, FGFR-1 AND PDGFR-β signaling in the cells. As expected, dose-dependence was found in the inhibitory effects of dovitinib on the phosphorylation of PDGFR-β, VEGFR-2, and FGFR-1, as well as their major downstream effector, the phosphorylation of ERK, on these cells (Figure 2C–D), but not the phosphorylation of Akt (Additional file 2: Figure S2B). While the levels of cleaved PARP and cleaved caspase 3 were also readily detected in dose-dependence of dovitinib (Additional file 2: Figure S2A).

### The proliferation of endothelial cells (but not the HCC cells) was inhibited by dovitinib

**Figure 3:**
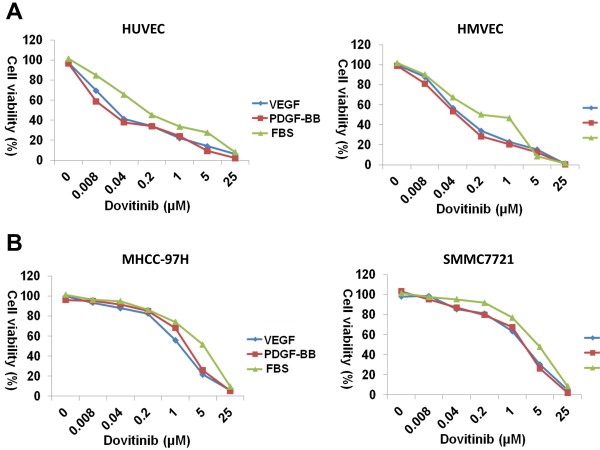
**Dovitinib inhibited the proliferation of endothelial cells at pharmacologically relevant concentration.A**) Dovitinib inhibited the proliferation of endothelial cells under VEGF, PDGF-BB dependent or normal conditions by MTS assay; results were normalized to DMSO controls. **B**) Proliferation of MHCC-97H and SMMC7721 HCC cells was inhibited only at high concentrations of dovitinib.

Only two HCC cell lines, MHCC-97H and SMMC7721, expressed PDGFR-β. Therefore, we compared the inhibitory effect of dovitinib on proliferation in these two lines and in endothelial cell lines. The IC50 for dovitinib to inhibit the proliferation of HCC cell lines was 0.87 ± 0.17 μmol/L and 1.26 ± 0.15 μmol/L for MHCC-97H and SMMC7721, respectively. While dovitinib showed robust inhibitory effect of endothelial cells under VEGF-dependent conditions were ~0.04 μmol/L, which was similar to the concentrations required to inhibit activation of VEGFR-2 (Figure 3). The IC_50_ values of MHCC-97H and SMMC7721 cells were much higher than that needed to inhibit the activation of PDGFR-β, suggesting that targeting of PDGFR-β by dovitinib did not influence the proliferation of these cells.

### Dovitinib inhibited the migration of endothelial cells but not of HCC cells

**Figure 4:**
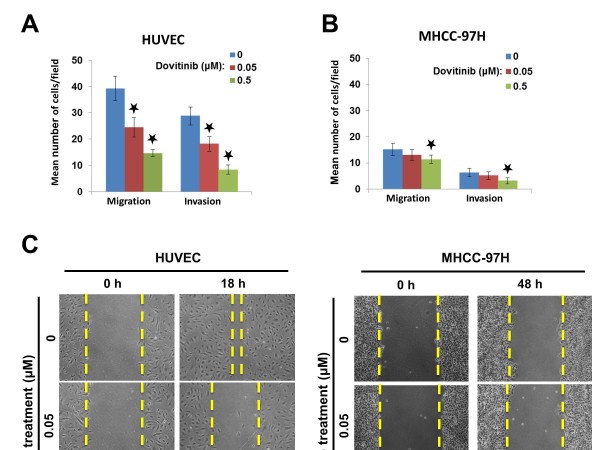
**Dovitinib inhibited the migration and invasion of endothelial cells at pharmacologically relevant concentrations.A**) As evaluated by Transwell assay, dovitinib significantly inhibited the migration and invasion of HUVEC endothelial cells in a dose-dependent manner. **B**) The migration and invasion of MHCC-97H cells was not inhibited by dovitinib. **C**) Wound healing assays showed the inhibitory effect of dovitinib on the migration of endothelial and HCC cells. Consistent with the results in the Transwell assay, the migration of HUVEC cells was inhibited, while MHCC-97H cells did not show motility. *Black star*, *P* < 0.01 relative to control cells.

Figure 4 shows that at pharmacologically relevant concentrations, dovitinib inhibited the migration and invasion of endothelial cells as evaluated by Transwell assay and wound-healing assay. The motility of MHCC-97H, SMMC7721 and QGY7703 was very weak in the wound-healing assay, and dovitinib did not show an significantly inhibitory effect on their migration of MHCC-97H.

### Dovitinib inhibited tumor angiogenesis in vivo

**Figure 5:**
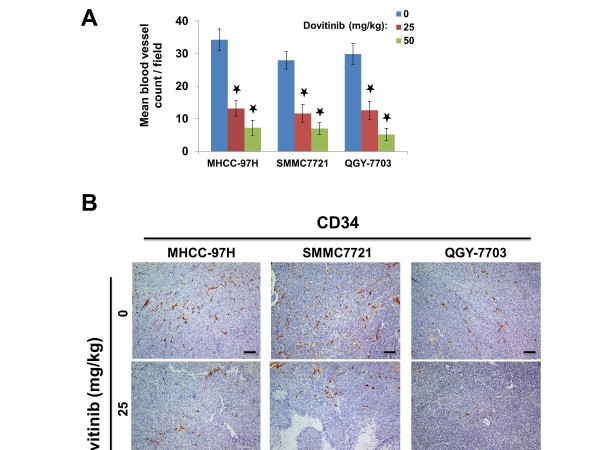
**Dovitinib inhibited tumor angiogenesis in vivo.A**) Treatment with dovitinib decreased microvessel density in a dose-dependent manner. *Black star*, *P* < 0.01 relative to the control group. **B**) Immunohistochemical staining of CD34 showing microvessels in xenograft tumors. Scale bars, 100 μm.

**Figure 6:**
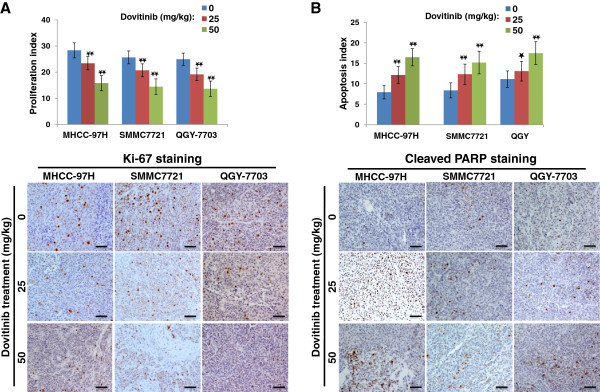
**Dovitinib inhibited proliferation and enhanced apoptosis in HCC xenograft tumors.A**) Ki-67 staining showed the proliferation of HCC cells was reduced in dovitinib-treated tumors. **B**) Cleaved PARP staining showed that apoptosis of HCC cells increased in dovitinib-treated tumors. Scale bars, 300 μm. *Black star*, *P* < 0.05; *double black star*, *P* < 0.01.

To further elucidate the mechanism of dovitinib-mediated inhibition of growth and metastasis in vivo, we collected xenograft tumor samples and examined the effect of dovitinib on the tumor vasculature as well as on HCC cell proliferation and apoptosis. Immunohistochemical analyses revealed that the markers of endothelial cell and pericyte expressed homogeneously in tumor sample (Additional file 3: Figure S3), and dovitinib significantly decreased microvessel density in MHCC-97H cells by 61.5% and 78.8% at doses of 25 mg/kg and 50 mg/kg, respectively; MVD was decreased by 58.3% and 74.8%, respectively, in SMMC7721 cells and by 57.9% and 82.6% in QGY-7703 cells (Figure 5). In comparison with the robust inhibition of angiogenesis, the effects of dovitinib on inhibiting proliferation and enhancing apoptosis of HCC cells in vivo were modest, although significant (Figure 6), suggesting that direct targeting HCC cells by dovitinib might not be the primary event inhibiting tumor growth in vivo.

## Discussion

Dovitinib is currently in Phase II studies for the treatment of advanced hepatocellular carcinoma (NCT01232296), but the underlying mechanism of dovitinib in targeting HCC is not known. Our results showed that dovitinib preferentially targeted endothelial cells by inhibiting their proliferation and motility and inhibiting angiogenesis in vivo. At pharmacologically relevant concentrations, dovitinib did not affect HCC cells.

Other groups have reported that dovitinib concentrations lower than 1 μmol/L are sufficient to inhibit RTK signaling [17,18]. In cellular assays, Andrew and colleagues found that dovitinib inhibited FGFR signaling in Ba/Fs cells lines of myeloproliferative syndrome with IC_50_ values of 0.09–0.15 μmol/L [28], consistent with our studies on endothelial cells. Finally, both clinical and preclinical pharmacodynamic studies showed that pharmacologically and clinically relevant plasma concentrations of dovitinib are 0.01–0.3 μmol/L [17,18].

A recent study using a high concentration (1.727 μM) of dovitinib reported that anchorage-independent growth and FGF-induced motility of HCC cells was inhibited [29]. Unfortunately, this study did not evaluate the effect of dovitinib on endothelial cells, and the concentration used was much higher than a pharmacologically relevant dose. In our study, dovitinib at 0.1 μmol/L did not affect the viability or proliferation of HCC cell lines in vitro. In contrast, it did inhibit endothelial cell proliferation and motility at concentrations that also inhibited VEGFR and FGFR signaling in these cells. Studies of HCC xenografts treated with pharmacologically relevant concentrations of dovitinib showed more effect on the inhibition of tumor angiogenesis in vivo than on proliferation or apoptosis. Taken together, these data indicate that dovitinib acts preferentially to target tumor vasculature rather than cancer cells in the treatment of HCC.

Some previous studies have reported that high expression of the angiogenic factors VEGF, basic FGF, and platelet-derived growth factor receptor are detected in patients with HCC, suggesting that VEGFR, FGFR, and PDGFR are likely targets of dovitinib. Our analysis of HCC and endothelial cell lines found that, of the known dovitinib-sensitive RTKs, only PDGFR-β was expressed by SMMC7721 and MHCC-97H cells, where VEGFR-2 and FGFR-1 were highly expressed by endothelial cells. Although high PDGFR-β expression has been correlated with HCC progression [30], our in vitro studies showed that dovitinib inhibition of PDGFR signaling was not sufficient to inhibit the proliferation of HCC cells. Thus, PDGFR signaling in HCC cells is likely through redundant growth signaling pathways. In contrast, dovitinib inhibited the phosphorylation of VEGFR-2 and FGFR-1 in endothelial cells at similar concentrations, indicating the important role of VEGFR and FGFR signaling in the proliferation of endothelial cells.

The endothelial cells recruited to the tumor tissue are not only related to blood perfusion of the tumor, but they are also believed to be involved in the cancer–stromal cell interaction favoring tumor growth [31]. However, some believe that normal endothelial cells may be the barrier to hematogenous metastasis [32]. Blocking the adhesion of tumor cells to endothelial cells prevents the implantation of tumors cells in capillaries, thus inhibiting the migration of tumor cells in the circulation [33]. In our previous study, we found in HCC tissues that a distinct pattern of endothelial structures, endothelium-coated tumor clusters, was an independent predictor for survival and recurrence in patients with HCC [6]. Endothelial cells facilitate the efficiency of metastasis, irrespective of the invasiveness of tumor cells [6]. In our study here, a pharmacologically relevant concentration of dovitinib, 0.1 μmol/L, did not affect the migration or invasion of HCC cell lines in vitro. In contrast, dovitinib did inhibit endothelial cell migration and invasion and did so at concentrations that also inhibited VEGFR and FGFR signaling in these cells. Moreover, studies of orthotopic HCC models treated with pharmacologically relevant concentrations of dovitinib inhibited lung metastasis in vivo. These findings suggest that the migration of endothelial cells could be an important step in HCC metastasis by providing an envelope that protects the tumor cells in circulation [6].

## Conclusion

In summary, we found that at pharmacologically relevant concentrations, dovitinib targeted endothelial cells, but not HCC cells, in inhibiting HCC growth and metastasis. To our knowledge, this is the first study to clarify the cellular target of dovitinib in the treatment of HCC, which might be helpful for future development of targeted therapy in HCC.

## Competing interests

The authors declare that they have no competing interests.

## Authors’ contributions

RPG and CNQ are responsible for the study design. ZYC, MS, and CNQ performed the experiments and draft the manuscript. LXP, XJL, ZXG, SHL, and CZ collected the data. ZYC, RPG, and CNQ participated in the data analysis and interpretation. All authors read and approved the final manuscript.

## The novelty and impact of the work

Dovitinib is a receptor tyrosine kinase (RTK) inhibitor in clinical trials for the treatment of hepatocellular carcinoma (HCC). We do not have a full understanding of the cellular target(s) of dovitinib in HCC treatment. By using five HCC cell lines and five endothelial cell lines in validating targets, we found that dovitinib inhibited HCC growth and metastasis preferentially through an antiangiogenic mechanism, not through direct targeting of HCC cells.

## Supplementary Material

Additional file 1 **Figure S1.** The markers of endothelial cell and pericyte expressed homogeneously in tumor sample. **A)** Immunohistochemical staining of CD31 in the tumor sample. **B)** CD34. **C)** CD105. **D)** SMA. Click here for file

Additional file 2 **Figure S2.** c-KIT and Flt-3 were undetectable in HCC cell lines and endothelial cell lines. Click here for file

Additional file 3 **Figure S3.** Effects of dovitinib on apoptosis and the phosphorylation of Akt in HCC cell and endothelial cell lines. **A)** The levels of cleaved PARP and cleaved caspase3 were also readily detected in dose-dependence of dovitinib, but it do not show significant difference between on HCC cell and endothelial cell lines. **B)** Dovitinib does not reduced the basal phosphorylation levels of Akt in HCCcell lines. Click here for file
