# Supplementary material for: Dovitinib preferentially targets endothelial cells rather than cancer cells for the inhibition of hepatocellular carcinoma growth and metastasis
Source: J Transl Med. 2012 Dec 10;10:245. doi: 10.1186/1479-5876-10-245 (PMC3552726; doi:10.1186/1479-5876-10-245)
Supplement: Additional file 1 — Figure S1. The markers of endothelial cell and pericyte expressed homogeneously in tumor sample. A) Immunohistochemical staining of CD31 in the tumor sample. B) CD34. C) CD105. D) SMA. [file 1479-5876-10-245-S1.ppt]

## Slide 1
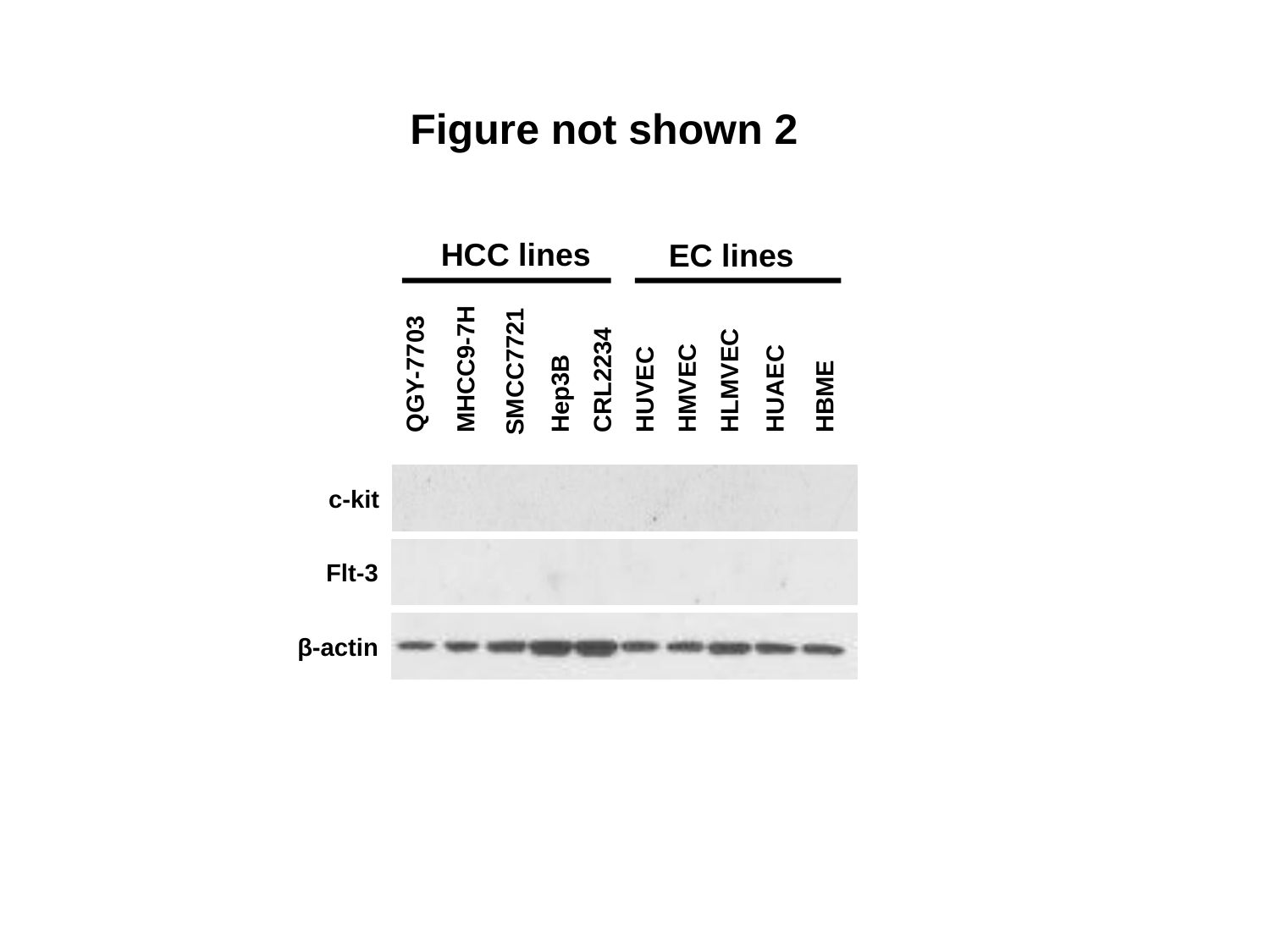

Figure not shown 2
HCC lines
EC lines
MHCC9-7H
SMCC7721
QGY-7703
HLMVEC
CRL2234
Hep3B
HUAEC
HUVEC
HMVEC
HBME
c-kit
Flt-3
β-actin
