# Supplementary material for: Dovitinib preferentially targets endothelial cells rather than cancer cells for the inhibition of hepatocellular carcinoma growth and metastasis
Source: J Transl Med. 2012 Dec 10;10:245. doi: 10.1186/1479-5876-10-245 (PMC3552726; doi:10.1186/1479-5876-10-245)
Supplement: Additional file 2 — Figure S2. c-KIT and Flt-3 were undetectable in HCC cell lines and endothelial cell lines. [file 1479-5876-10-245-S2.pptx]

## Slide 1
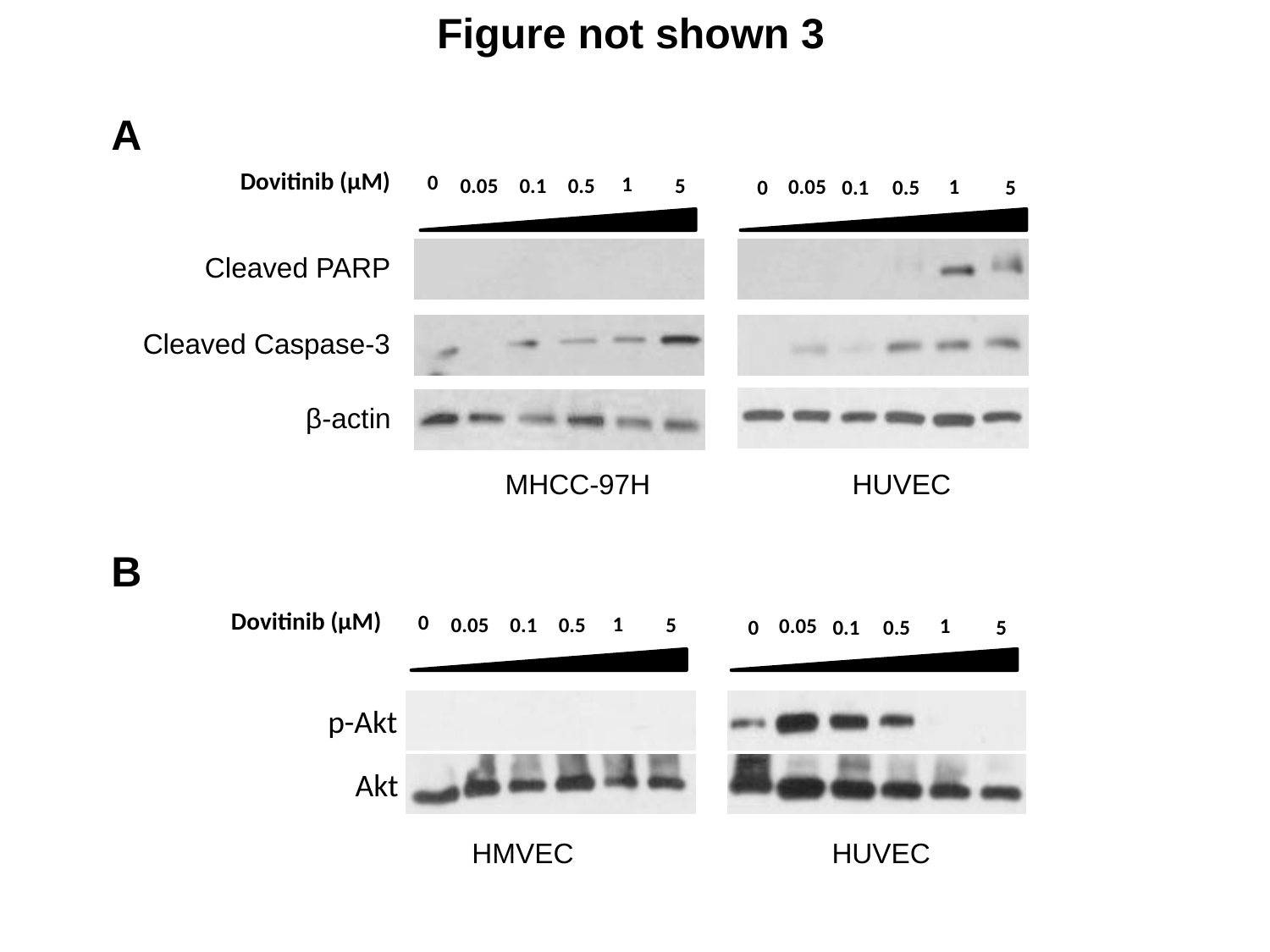

Figure not shown 3
A
Dovitinib (μM)
0
1
0.05
0.1
0.5
5
0.05
1
0
0.1
0.5
5
Cleaved PARP
Cleaved Caspase-3
β-actin
MHCC-97H
HUVEC
B
Dovitinib (μM)
0
1
0.05
0.1
0.5
5
0.05
1
0
0.1
0.5
5
p-Akt
Akt
HMVEC
HUVEC
