# Supplementary figures and images for: Dovitinib preferentially targets endothelial cells rather than cancer cells for the inhibition of hepatocellular carcinoma growth and metastasis
Source: J Transl Med. 2012 Dec 10;10:245. doi: 10.1186/1479-5876-10-245 (PMC3552726; doi:10.1186/1479-5876-10-245)

## Slide 1
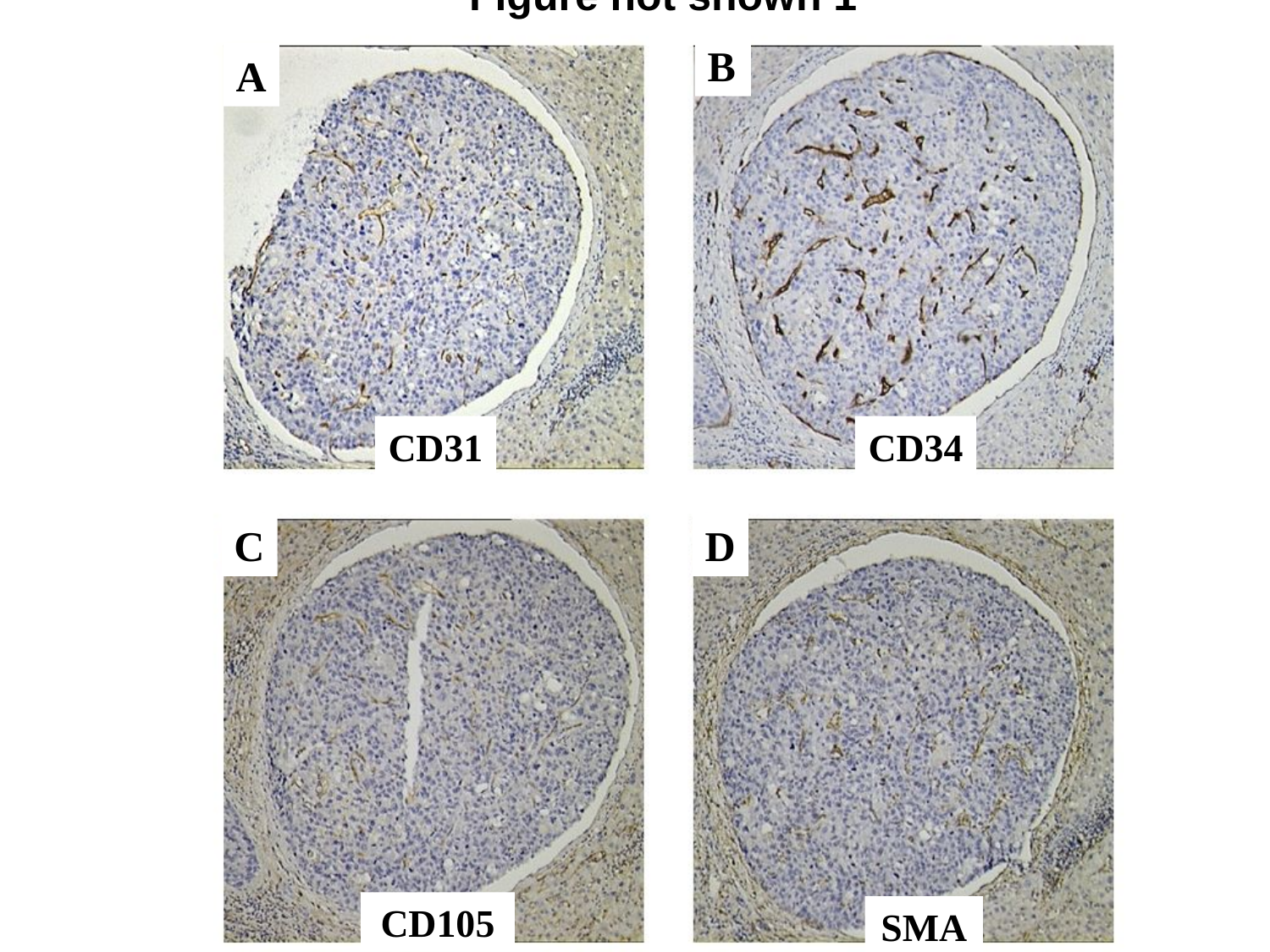

Figure not shown 1
B
A
CD31
CD34
C
D
CD105
SMA

Supplement: Additional file 3 — Figure S3. Effects of dovitinib on apoptosis and the phosphorylation of Akt in HCC cell and endothelial cell lines. A) The levels of cleaved PARP and cleaved caspase3 were also readily detected in dose-dependence of dovitinib, but it do not show significant difference between on HCC cell and endothelial cell lines. B) Dovitinib does not reduced the basal phosphorylation levels of Akt in HCCcell lines. [file 1479-5876-10-245-S3.ppt]
